# Process-Related Incidents in Nuclear Medicine: A Four-Year Single-Center Retrospective Analysis to Support the Implementation of a Scenario-Based Radiopharmacy Training

**DOI:** 10.3390/pharmacy14010032

**Published:** 2026-02-10

**Authors:** Yasmine Soualy, Stéphane C. Renaud, Jade Torchio, Juliette Fouillet, Julie Ensenat, Léa Rubira, Cyril Fersing

**Affiliations:** 1 Nuclear Medicine Department, Institut Régional du Cancer de Montpellier (ICM), University Montpellier, 34298 Montpellier, France; 2 IBMM, University Montpellier, CNRS, ENSCM, 34293 Montpellier, France

**Keywords:** nuclear medicine, good radiopharmacy practice, radiopharmaceuticals, process-related incident, quality of healthcare, quality assurance, scenario-based training

## Abstract

Nuclear medicine is a medical specialty combining parenteral radioactive drug handling and complex clinical workflows, making systematic process-related incident (PRI) analysis essential to support healthcare quality improvement. This study reports a four-year single-center retrospective analysis of PRIs in a nuclear medicine department and describes the development and implementation of a scenario-based radiopharmacy training program for nuclear medicine technologists (NMTs) derived from these findings. PRIs were extracted from the institutional reporting system and categorized according to a structured classification. Training scenarios were designed from recurrent radiopharmacy-related PRIs, and their impact was evaluated using a knowledge questionnaire administered pre and post training. A total of 223 PRIs were analyzed, of which 38.6% (n = 86) were related to the radiopharmaceutical circuit. Among these, 28.3% occurred exclusively within the radiopharmacy cleanroom. Administration (19%), dispensing (15%), delivery and reception (15%), and preparation and quality control (15%) of radiopharmaceuticals were the most frequently involved stages. No PRI exceeded a moderate criticality level. Eight NMTs participated in the training program, consisting of an analysis of videos depicting the developed scenarios. The mean knowledge score increased significantly from 7.51/10 before training to 8.46/10 four weeks after training (*p* = 0.02), with marked improvements in hygiene- and radioactivity-related topics. These results support the use of retrospective PRI analysis as an operational basis for specific, scenario-based training to strengthen safety practices in radiopharmacy settings.

## 1. Introduction

Nuclear medicine (NM) is a medical specialty that integrates radiopharmaceutical production, the use of ionizing radiation, complex clinical workflows, and multidisciplinary collaboration [1,2]. This medical specialty becomes particularly high-risk as soon as therapeutic procedures are implemented, including metabolic radiotherapy or targeted radionuclide therapy [3]. The sterile compounding, dispensing, and administration of radiopharmaceuticals involve stringent pharmaceutical and radiation protection requirements, as well as time-sensitive constraints related to radionuclide physical decay [4]. In this context, quality assurance systems are essential to ensure the safety, efficacy, and traceability of diagnostic and therapeutic procedures, while minimizing risks for patients, healthcare professionals, and the environment [5].

Quality assurance in NM relies on the application of structured organizational frameworks encompassing good pharmaceutical practices, radiation protection regulations, and institutional risk management policies. In particular, radiopharmacy activities represent a critical component of the radiopharmaceutical circuit and are subject to reinforced regulatory oversight [6]. These activities combine aseptic preparation requirements with constraints specific to the handling of unsealed radioactive sources, thereby increasing procedural complexity and exposure to potential failures. Any deviation in radiopharmaceutical preparation, quality control, dispensation, or timing of administration may affect biodistribution, image quality, dosimetry, or therapeutic efficacy, with direct consequences for the patient [7].

Process-related incidents (PRIs) in NM may occur at any stage of the radiopharmaceutical circuit, from supply and reception of radioactive materials to waste management. In radiopharmacy practice, supply chain disruptions, delays in radiopharmaceuticals delivery, or generator failures may compromise scheduling and continuity of care [8]. During reception and control of radioactive packages, non-conformities related to documentation, activity calibration, contamination checks, or traceability may generate radioprotection risks and operational delays. The aseptic preparation of radiopharmaceuticals constitutes a particularly critical step, as it combines pharmaceutical, microbiological, and radiological hazards [9,10]. Deviations in environmental conditions, operator technique, equipment performance, or quality control procedures may lead to non-compliant products, increased occupational exposure, or potential harm to patients [11,12].

Dispensing and administration further represent high-risk interfaces between radiopharmacy and clinical practice. Errors related to patient identification, radiopharmaceutical selection, activity calculation, or injection timing may result in misadministration, suboptimal imaging, or unintended radiation exposure [13,14,15,16]. These risks are exacerbated by workload, time pressure, and the need for precise coordination between radiopharmacists, NM physicians, and nuclear medicine technologists (NMTs) [17]. Consequently, NM departments are particularly vulnerable to both PRIs associated with care and near-miss events that reveal latent system weaknesses [18].

The structured reporting of PRIs is therefore a cornerstone of quality management and risk control in NM [19]. Incident reporting systems provide a formal mechanism to capture deviations, near misses, and adverse outcomes, enabling traceability and regulatory compliance while supporting organizational learning. Importantly, modern approaches to patient safety emphasize a non-punitive culture of reporting, in which PRIs are considered indicators of system vulnerabilities rather than individual failures [20,21]. This paradigm shift facilitates voluntary reporting and contributes to the development of a mature safety culture within healthcare organizations.

Nevertheless, the effectiveness of PRI reporting depends on the subsequent analysis and use of collected data. Retrospective analysis of PRIs allows identification of recurrent failure modes, critical process steps, and contributing factors, including technical, human, and organizational determinants [19,22]. In the specific context of NM, such analyses are essential to prioritize risk mitigation strategies, given the multiplicity of regulatory constraints and the high stakes associated with radiopharmaceutical use. As a complement to registry studies [23,24,25,26,27], single-center retrospective studies are particularly valuable, as they account for local workflows, infrastructure, and resource constraints that may influence the occurrence and nature of PRIs [19,28].

Translating PRI analysis into effective preventive actions remains a major challenge in healthcare risk management. Among available strategies, targeted training interventions play a central role in reinforcing both technical and non-technical skills [29,30]. Scenario-based training has emerged as a particularly relevant educational approach in high-risk environments, as it enables experiential learning based on realistic situations derived from actual incidents [31]. By reproducing critical scenarios identified through PRI analysis, this method facilitates understanding of error mechanisms, enhances situational awareness, and promotes appropriate decision-making under operational constraints.

For NMTs, who occupy a pivotal position at the interface between radiopharmacy operations, imaging systems, and direct patient care, scenario-based training is especially relevant [32,33]. Their role involves not only technical execution of procedures but also adherence to asepsis, hygiene and radiation protection principles, verification of patient identity and radiopharmaceutical conformity, and timely communication with other professionals. Training programs grounded in real PRIs may therefore address both procedural compliance and transversal competencies such as communication, teamwork, and risk anticipation, contributing to a more robust safety culture within NM departments.

In this context, the present study reports a four-year single-center retrospective analysis of PRIs reported in a NM department, with a specific focus on radiopharmacy-related incidents. This initial work characterized the nature, distribution, and contributing factors of reported events in order to support the design and implementation of a scenario-based small-group training program tailored to NMTs involved in radio-pharmaceuticals preparation. This approach illustrates how systematic analysis of PRIs can be leveraged as an operational tool to strengthen quality assurance, enhance professional training, and improve safety across the radiopharmaceutical circuit.

## 2. Materials and Methods

### 2.1. Retrospective Analysis of PRIs

#### 2.1.1. PRI Reporting System and Data Collection

PRIs were identified through the institutional internal reporting system Ennov^®^ Document (Ennov Solutions, Paris, France), a centralized and secured electronic platform accessible to all healthcare professionals of the institution. This system enables direct reporting of any incident, near miss, or PRI encountered during routine clinical, technical or organizational activities. PRI reporting is based on a standardized electronic form designed to collect comprehensive information required for subsequent analysis. Reported data include the date and time of occurrence, unit, clinical or organizational context, estimation of frequency and severity, immediate corrective actions, functional identity of the reporter, and, when applicable, identifiers of the persons involved, including the patient’s permanent identification number. The scope of reported events in NM covers incidents involving patients, radiopharmaceuticals, medical devices, technical procedures, organizational failures, equipment malfunctions, supply chain disruptions, or interactions between professionals or with patients.

All PRIs reported within our NM department between January 2021 and December 2024 constituted the dataset for this study. Data were extracted into an Excel^®^ file by the department’s quality officer and anonymized prior to analysis.

#### 2.1.2. Categorization of Adverse Events

**Figure 1:**
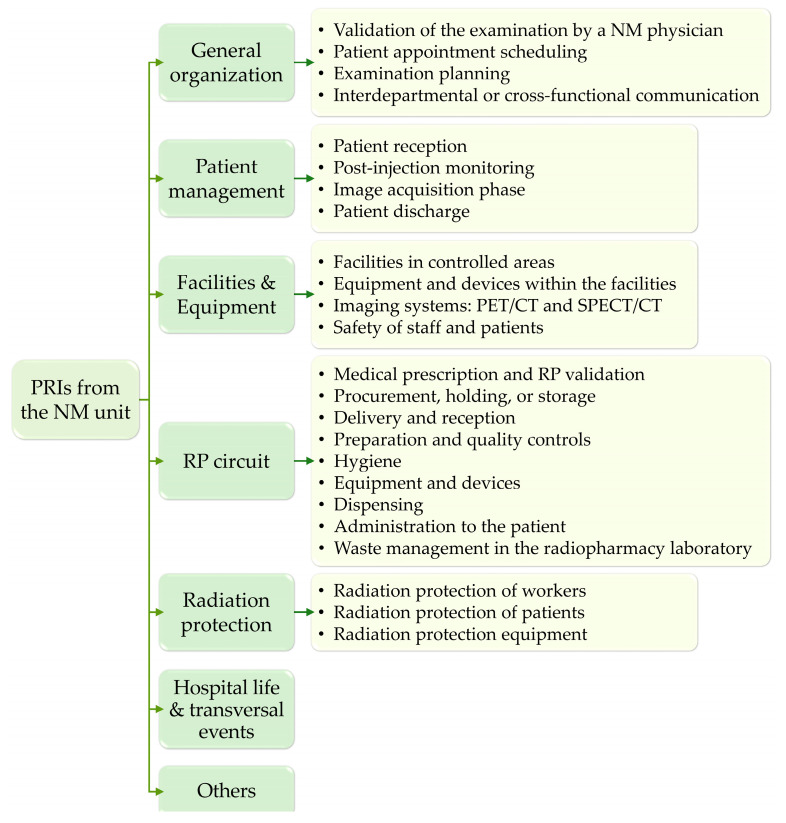
Classification of categories (green) and subcategories (yellow) used to organize the PRIs. PET = positron emission tomography; SPECT = single-photon emission computed tomography; CT = computed tomography; RP = radiopharmaceutical.

To enable homogeneous analysis and obtain a comprehensive overview of the vulnerabilities of our NM department, a structured classification system was developed around 7 categories and 24 subcategories, reflecting both the patient care pathway and the radiopharmaceutical circuit (Figure 1). All PRI reports were independently reviewed by a working group composed of three radiopharmacists, one quality officer and one radiopharmacy resident. Complex events involving multiple failure modes were split into distinct reports when they corresponded to different categories. PRI categories included (1) general organization; (2) patient management; (3) facilities and equipment outside the radiopharmacy laboratory; (4) radiopharmaceutical circuit; (5) radiation protection; (6) hospital life and transversal events; and (7) a residual “others” category. The radiopharmaceutical circuit category encompassed prescription and radiopharmaceutical validation, supply, reception, preparation, quality control, dispensing, administration, and waste management.

Each PRI was assigned to a category and a subcategory (with the exception of events classified as hospital life and transversal events and others) in order to provide a detailed characterization of each event.

#### 2.1.3. Distribution of PRIs by Professional Role and Activity Sector

Each PRI was associated with the professional role of the initial reporter, grouped into 8 categories: pharmacist, NMT, physician, medical physicist, manager, medical assistant, medical laboratory technician, and healthcare assistant.

Events were also classified according to the sector of activity in which they occurred. To harmonize this distribution, 4 main sectors were defined: conventional NM (single-photon emission tomography, SPECT), positron emission tomography (PET), therapy, and a residual “others” category. PRIs related to SPECT, PET, and therapy could occur either within the NM department or within the radiopharmacy laboratory.

#### 2.1.4. PRIs Criticality Assessment

PRI criticality was assessed using a simplified linear risk matrix, combining estimated frequency of occurrence and severity of consequences [34]. Frequency was graded on a five-level scale, ranging from exceptional (1) to daily occurrence (5). Severity was also graded on a five-level scale and estimated based on actual or potential impact on patient safety, staff safety, or organizational functioning, ranging from minor (1) to potentially lethal (5) (Table 1).

Each PRI was positioned within the severity–frequency matrix, generating a criticality score calculated as severity × frequency, with values ranging from 1 to 25. Risk levels were classified as low (1–4), moderate (5–9), high (10–16), or critical (20–25). Initial scoring was performed by the quality management team and subsequently reviewed and harmonized by the radiopharmacy-driven working group to ensure consistency across all analyzed events.

Overall, a descriptive analysis of PRIs was conducted according to the following parameters: year of occurrence, professional role of the reporter, activity sector of occurrence, criticality, and PRI category and subcategory.

### 2.2. Design of a Scenario-Based Radiopharmacy Training Program

#### 2.2.1. Development of the Training Project

The scenario-based radiopharmacy small-group training program was developed by the same working group previously involved in the retrospective analysis of PRIs. Meetings were held to define the educational objectives, select appropriate pedagogical modalities, and ensure alignment between the PRI analysis and the proposed training content. Progress monitoring and collective discussion allowed refinement of the training structure and validation of its feasibility.

Based on the analysis of PRIs related to the radiopharmaceutical circuit, four major subcategories were selected: delivery and reception, preparation and quality controls, dispensing, and hygiene. For each subcategory, the most frequently reported PRIs were identified and used as the basis for scenario development. Scenarios were intended to represent routine laboratory situations and to emphasize recurrent deviations observed in practice. Such a visual and immersive approach was deliberately favored over written descriptions, as it was considered more effective in capturing attention and promoting long-term retention [35,36].

#### 2.2.2. Filming of the Scenario-Based Videos

All materials required for scenario setup were progressively identified and collected during a preparation phase. For radiation protection purposes, all scenes were recorded without radioactive materials, using containers filled exclusively with saline solution. Filming was performed outside routine laboratory activity to avoid any interference with clinical operations.

A total of seven videos were filmed, each corresponding to a single scenario simulating between two and six technical or organization errors. The scenarios were enacted by a radiopharmacist, while the radiopharmacy resident was responsible for filming and editing (iMovie, Apple Inc., Cupertino, CA, USA). The videos were designed to be concise, realistic, and clearly structured to facilitate observation, identification and discussion during training sessions.

#### 2.2.3. Development of the Knowledge Assessment Questionnaire and the Training Satisfaction Survey

A knowledge assessment questionnaire was developed in parallel with the training scenarios to evaluate the educational impact of the program. Questions were in direct correspondence with the videos scenario and PRI analysis, focusing on radiopharmacy cleanroom, hygiene, good radiopharmacy practices (GRPs), and radioactivity. The questionnaire included both single- and multiple-choice questions. To maintain consistency with the interactive nature of the training, the questionnaire was administered using the Wooclap^®^ online platform (Brussels, Belgium) and was completed via participants’ mobile devices. A predefined scoring system allowed partial credit according to the number of incorrect answers, enabling a detailed assessment of baseline knowledge and subsequent progression following the training. The scoring system applied was as follows: 1 point for a fully correct answer (single-choice questions scored binarily as 0 or 1), 0.5 points for 1 error, 0.2 points for 2 errors, and 0 points for more than 2 errors.

A satisfaction survey assessing the training program as a whole was developed and administered to participants at the end of the training program, to be completed anonymously. The questionnaire comprised seven items rated on a five-point Likert scale and two open-ended short-answer questions. Three items assessed the training format (overall satisfaction, organization, and quality of interactions), one item evaluated the duration of the training, and three items focused on content-related aspects (knowledge gain, perceived difficulty, and ease of application in practice). The two open-ended questions invited participants to propose additional training topics and to suggest areas for improvement. Response scales varied according to the nature of the item: for most items, scores ranged from 0 (very unsatisfactory) to 5 (very satisfactory); for perceived difficulty, from 0 (very difficult) to 5 (very easy); and for ease of application, from 0 (not comfortable at all) to 5 (very comfortable).

#### 2.2.4. Organization and Evaluation of Training Sessions

The training was designed exclusively for NMTs authorized to work in the radiopharmacy cleanroom. Participants were not informed in advance of the training format or content in order to maximize pedagogical impact and limit anticipation bias.

**Figure 2:**
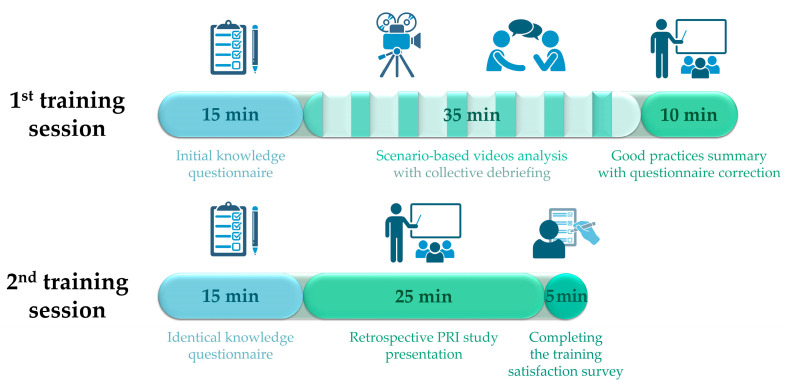
Timelines of the two training sessions.

Two training sessions were organized (Figure 2). The first session, lasting one hour, included the initial knowledge questionnaire followed by scenario-based videos analysis with collective debriefing and reminders of good practices. The second session, lasting 45 min and organized four weeks later, consisted of a repeat of the knowledge questionnaire under identical conditions to assess knowledge retention. This session also included a presentation of aggregated results from the four-year retrospective PRI analysis to promote quality culture and emphasize the importance of PRI reporting. Participants were subsequently invited to complete the training satisfaction survey.

Analysis of the knowledge questionnaires was based on individual scores, with calculation of means and medians. Statistical comparison between pre- and post-training results was performed using a one-sided paired Student’s *t*-test, after confirmation of distribution normality using the Shapiro–Wilk test (*p* = 0.841) and verification of variance homogeneity using Fisher’s *F*-test (*p* = 0.326).

## 3. Results

### 3.1. Retrospective Analysis of PRIs

#### 3.1.1. Distribution of Reported PRIs by Year

**Figure 3:**
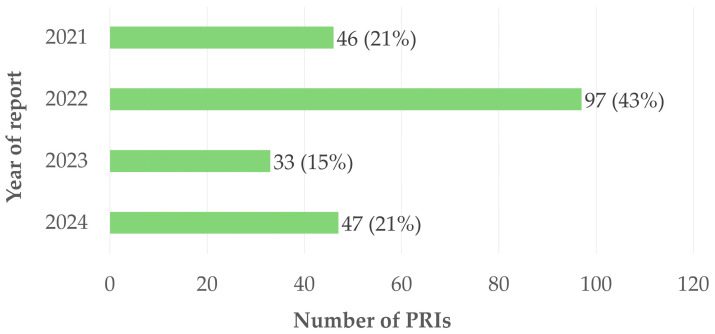
Distribution of the number of PRIs by year.

Between 2021 and 2024, 187 reports were recorded in the NM department through the internal reporting system. Among these reports, eleven complex cases were split into two distinct events, two were split into three events, and two were split into four events, resulting in a total of 223 individual PRIs. Figure 3 shows a marked increase in the number of reported events in 2022, with 97 PRIs, compared with 46 in 2021, 33 in 2023, and 47 in 2024. This increase in 2022 can be attributed to facility renovation of the radiopharmacy cleanroom, which led to temporary reorganization of activities, adaptation of procedures, and operating conditions potentially associated with a higher level of risk, thereby favoring the occurrence or detection of adverse events.

#### 3.1.2. Distribution of PRIs According to the Reporter’s Role and Activity Sectors

**Figure 4:**
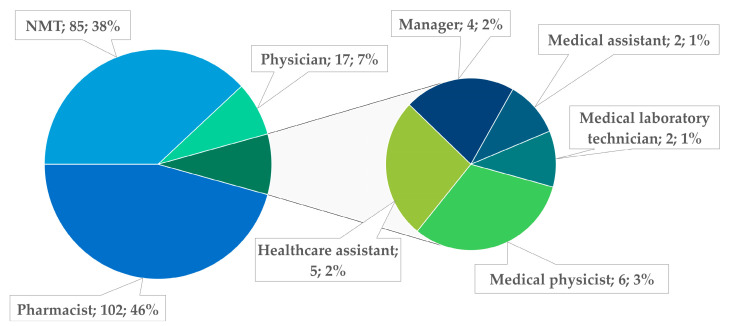
Distribution of PRIs (number; proportion) by the reporter’s role.

A high representation of pharmacists (46%) and NMTs (38%) is observed among the healthcare professionals reporting PRIs (Figure 4), reflecting their strong involvement in PRI reporting. This trend can be explained by their central role in the radiopharmaceutical circuit, their direct exposure to highly technical steps, and a well-established quality culture embedded in their routine practices. In contrast, NM physicians (8%), medical physicists (3%), and other staff categories appear to be less involved, suggesting an uneven adoption of the reporting system. This disparity highlights the need for improved awareness among specific professional groups and for promoting the integration of PRI reporting into their daily practice.

The distribution of PRIs according to the activity sectors of the NM department reveals a marked concentration of reports in the SPECT (52%) and PET (41%) areas (Table 2). This predominance can largely be attributed to the higher workload in these sectors. The succession of technical steps, the need for close coordination between professionals, the logistical constraints associated with radiopharmaceuticals, and strict time requirements increase process vulnerability, thereby exposing these areas to a higher risk of failures. Therapy represents the highest-risk activity within the department, accounting for 6% of reported PRIs. The relatively small number of PRIs reported in therapy can be interpreted in different ways: first, this activity represents the smallest number of patients (1268 in 2021–2024 vs. 17,021 in PET and 13,940 in SPECT). Second, it may reflect strong control of protocols and heightened vigilance by the teams, or it may indicate potential underreporting. As even minor errors in therapeutic procedures can have significant consequences for patients or staff, raising awareness among professionals involved in this activity is essential.

#### 3.1.3. Distribution of PRIs According to Their Criticality, Categories and Subcategories

**Figure 5:**
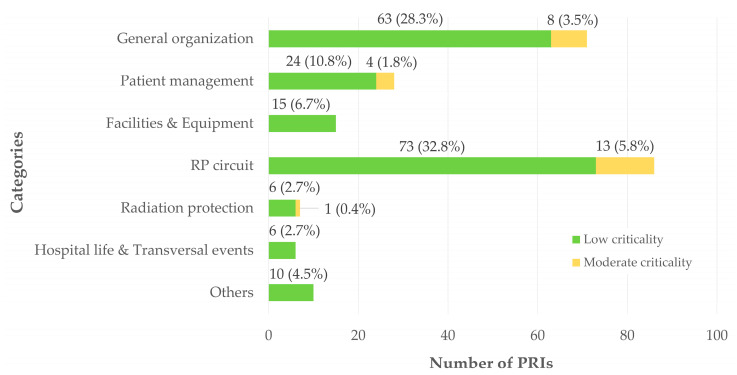
Distribution of PRIs (number; proportion) according to the main categories of the classification and to the criticality. Proportions were calculated based on the total number of PRIs analyzed (n = 223).

The distribution of PRIs according to their categorization is illustrated in Figure 5. General organizational issues accounted for 31.8% of reported adverse events (n = 71). These events were mainly related to appointment scheduling (16.1%, n = 36) and examination planning (11.2%, n = 25).

PRIs related to patient management represented 12.6% of cases (n = 28) and occurred at various stages of the patient care pathway. Most of these events took place during image acquisition under the camera (7.6%, n = 17), while others occurred during patient reception (3.1%, n = 7), post-injection monitoring (1.3%, n = 3), or at patient discharge from the department (0.5%, n = 1).

PRIs associated with facilities and the environment outside the radiopharmacy cleanroom accounted for 6.7% (n = 15). These were primarily due to malfunctions of equipment and devices (4.9%, n = 11), with fewer events related to safety issues (0.9%, n = 2), camera-related malfunctions (0.45%, n = 1), or facility-related issues (0.45%, n = 1).

The radiopharmaceutical circuit was the most represented category, accounting for 38.6% of PRIs (n = 86). These events were distributed across all stages of the circuit, including administration (7.2%, n = 16), dispensing (5.8%, n = 13), delivery and receipt (5.8%, n = 13), preparation and quality control (5.8%, n = 13), and prescription or pharmaceutical validation (4.5%, n = 10). Additional events were related to hygiene issues (3.6%, n = 8), equipment and devices (2.3%, n = 5), and procurement, holding, or storage (3.1%, n = 7).

Radiation protection-related PRIs accounted for 3.1% of reports (n = 7). They were equally divided between equipment issues (1.3%, n = 3) and occupational radiation protection (1.3%, n = 3), with a single PRI related to patient radiation protection (0.5%, n = 1).

Transversal situations related to hospital life represented 2.7% of adverse events (n = 6), while 4.5% of reports (n = 10) could not be assigned to a specific category.

No recorded PRI exceeded a criticality score of 9, corresponding to a moderate level of risk. The absence of reported high- or critical-severity PRIs suggests that nuclear medicine and radiopharmacy practices are effectively secured, likely reflecting the robustness of the quality management system in place. The categories accounting for the highest proportions of PRIs with moderate criticality were the radiopharmaceutical circuit (5.8% of total PRIs), general organization (3.5%), and patient management (1.8%). Notably, the radiopharmaceutical circuit exhibited the highest proportion of moderate-criticality events, with 15.1% of reports within this category classified as moderate in severity.

**Figure 6:**
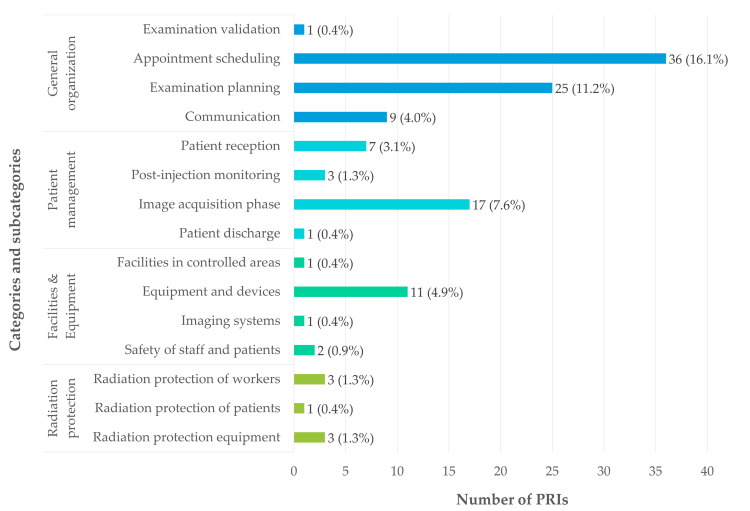
Distribution of PRIs according to classification subcategories, excluding radiopharmaceutical circuit. Proportions were calculated based on the total number of PRIs analyzed (n = 223).

The subcategories involved (except the radiopharmaceutical circuit) are presented in Figure 6, highlighting the predominance of organizational PRIs, largely driven by appointment scheduling and examination planning, which together account for the largest proportion of reported events. Nevertheless, the distribution of PRIs indicates that the radiopharmaceutical circuit is the most frequently involved category (n = 86, 38.6%), and it is therefore examined in detail in the following section.

#### 3.1.4. Detailed Analysis of PRIs Relating to the Radiopharmaceutical Circuit

**Figure 7:**
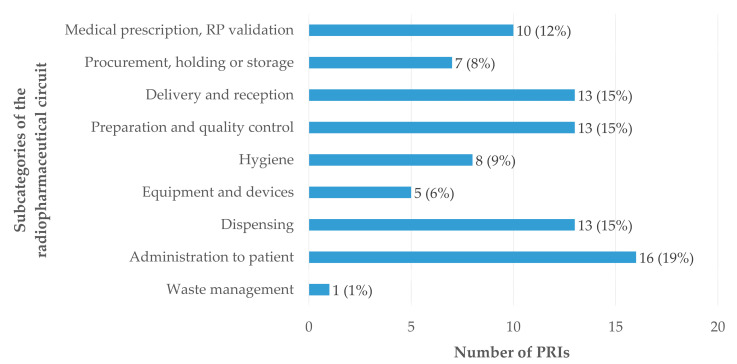
Distribution of PRIs related to the radiopharmaceutical circuit, according to the subcategories of the classification. Proportions were calculated based on the total number of PRIs within the RP circuit category (n = 86).

The analysis of the 86 PRIs occurring within the radiopharmaceutical circuit shows a relatively homogeneous distribution across the different subcategories (Figure 7). Administration of radiopharmaceuticals was the most frequently represented stage, accounting for 19% (n = 16) of reported events, highlighting the need for increased vigilance and effective training at this critical step. The dispensing, delivery and receipt, and preparation and quality control stages, each involved in 15% (n = 13) of PRIs, also represent particularly vulnerable steps. The preparation and quality control and dispensing steps require rigorous coordination and strict adherence to GRP. Errors related to delivery and reception logistics highlight the importance of secure reception procedures and appropriate training of the professionals involved. Operations related to procurement, holding, and storage account for 8% (n = 7) of PRIs and expose the system to risks of supply disruptions or ordering errors, which may compromise radiopharmaceutical availability and examination scheduling. Hygiene-related adverse events, representing 9% (n = 8) of reports, underscore the critical need to comply with GRP, particularly in an environment combining aseptic handling and radiation protection constraints. Hygiene audits and regular reminders of best practices may help reduce their occurrence. Finally, incidents related to equipment and devices (6%, n = 5) and to the environment (1%, n = 1), although less frequent, justify the implementation of rigorous preventive maintenance programs and optimization of internal logistical workflows. Overall, the medication circuit requires enhanced monitoring and vigilance.

Importantly, of the 223 reported PRI reports, 28.3% (n = 63) occurred exclusively within the radiopharmacy cleanroom, highlighting the relevance of the activities performed in this setting as key targets for focused training interventions.

### 3.2. Scenario-Based Radiopharmacy Training Program

#### 3.2.1. Production of Videos and Associated Materials

The seven videos developed from the predefined scenarios were designed with the educational objective of familiarizing NMTs working in the radiopharmacy cleanroom, with the main PRIs reported over the previous four years, while raising awareness of GRP aimed at reducing their risk of occurrence.

Videos were filmed within the radiopharmaceutical preparation laboratory during two sessions following routine activities of the unit, representing a total preparation and filming time of 3 h. Table 3 summarizes the theme and content of each video. The educational videos are available in the Videos S1−S7.

The knowledge assessment questionnaire submitted to training participants at the beginning of both sessions is available in the File S1. It included ten questions, with seven single-choice questions and three multiple-choice questions.

Test of the training program under simulated conditions validated the structuration in two sessions, as presented in Figure 2.

#### 3.2.2. Knowledge Questionnaire Results

**Figure 8:**
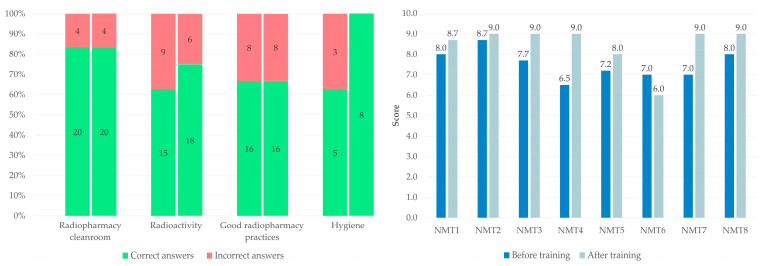
Results of the knowledge questionnaire administered before and after the training, showing the number and proportion of correct and incorrect responses by topic (**left**) and the individual scores obtained by each nuclear medicine technologist (**right**).

The pre-training questionnaire, comprising 10 questions distributed across four thematic areas (radiopharmacy cleanroom, n = 3; radioactivity, n = 3; GRP, n = 3; hygiene, n = 1), was administered to eight NMTs, resulting in a total of 80 responses analyzed. For each thematic area, the number and percentage of incorrect responses were calculated and are presented in Figure 8. Radioactivity, hygiene, and GRP themes exhibited the highest proportions of incorrect answers (38%, 38% and 33%, respectively), highlighting specific areas of vulnerability prior to the training. The same questionnaire was subsequently re-administered to the same participants four weeks after the training. The results demonstrated no improvement in the radiopharmacy cleanroom and GRP domains, a marked improvement in hygiene with a complete absence of incorrect responses during the second session, and a reduction in error rates in the radioactivity theme (from 38% to 25%). These findings suggest a positive mid-term impact of the scenario-based training on participants’ knowledge in the evaluated domains.

The overall mean pre-training score was 7.51/10, with a median score of 7.45/10, reflecting a generally balanced distribution of results and an overall satisfactory baseline level of knowledge. Individual scores ranged from 6.5/10 to 8.7/10. The mean post-training score increased from 7.51/10 to 8.46/10 (*p* = 0.02), with a median of 9.0/10, indicating that most participants (P1 to P8) achieved high scores, ranging from 6.0/10 to 9.0/10 (Figure 8). Participant 6 was the only operator to obtain a lower score during the second session.

#### 3.2.3. Results of the Training Satisfaction Survey

Overall satisfaction with the training was high, with a mean satisfaction score of 4.52/5. Mean scores by category were 4.71/5 for the training format, 4.50/5 for duration, and 4.33/5 for content. Of the eight participants, three provided responses to the two open-ended questions included at the end of the satisfaction survey. One participant suggested introducing theoretical training on investigational medicinal products prior to their implementation in the department. Two participants expressed a desire for more time allocated to discussion and recommended avoiding scheduling training sessions during the lunch break. The training satisfaction survey is available in the File S2.

## 4. Discussion

Given the highly specialized nature of NM activities, maintaining safety throughout the patient care pathway and the radiopharmaceutical workflow represents a constant and central challenge in daily practice [37]. The combination of complex clinical processes, strict regulatory requirements, multidisciplinary coordination, and the handling of unsealed radioactive sources exposes NM departments to a wide range of potential failures. In this context, structured PRI reporting systems play a key role in identifying vulnerabilities and supporting continuous improvement in patient and staff safety [19,38]. Also, the International Atomic Energy Agency (IAEA) framework on safety culture in nuclear medicine emphasizes the role of organizational, human, and procedural factors in ensuring safety [39].

Our study, which analyzes situations in close alignment with internationally recognized safety and risk management approaches promoted by the IAEA, provides findings that are consistent with previous investigations conducted in nuclear medicine settings. A recent analysis of safety events reported through an institutional incident notification system showed that medication-related incidents were the most frequently reported, followed by errors associated with clinical administration and procedural issues during clinical workflows [19]. A similar distribution was observed in our department, where PRIs related to the radiopharmaceutical circuit represented the largest proportion of reports. These findings highlight the centrality of medication-use processes in NM safety, while not overlooking the need for comprehensive safety strategies addressing all stages of patient management.

Within this framework, the radiopharmaceutical circuit emerges as a particularly critical area. Its technical complexity, high level of specialization, and essential role in diagnostic and therapeutic procedures make it intrinsically vulnerable to deviations [6]. Our detailed analysis revealed that PRIs were distributed across all stages of the circuit, including delivery and reception, preparation and quality control, dispensing, and administration. This relatively homogeneous distribution suggests that risks are not confined to a single step but rather reflect systemic vulnerabilities requiring transversal and regular corrective actions [6]. While several studies have addressed non-conformities in radiopharmacy, most have focused primarily on analytical or physicochemical quality controls of radiopharmaceuticals [40,41], whereas our findings underline the importance of organizational and human factors throughout the entire process.

The high proportion of PRIs occurring within the radiopharmacy cleanroom further supports the need for targeted preventive actions in this setting. As a specific aspect of pharmaceutical sterile compounding, radiopharmacy activities combine aseptic preparation requirements with radiation protection constraints, creating an environment in which deviations related to hygiene, workflow organization, or handling practices may have compounded consequences [42,43,44]. Although compliance with good manufacturing practices (GMPs) is mandatory and essential [45], our results suggest that regulatory frameworks alone may not fully prevent incidents related to human factors, workload, or situational awareness. This observation reinforces the importance of complementary approaches aimed at strengthening professional practices and safety culture.

In this respect, the scenario-based training program developed in our study represents a pragmatic translation of PRI analysis into concrete preventive action. Scenario-based education has been widely recognized as an effective pedagogical approach in high-risk healthcare environments, as it promotes experiential learning, improves error detection, and fosters reflection on both technical and non-technical skills [46,47]. By grounding training scenarios directly in real PRIs encountered in our department, the program sought to maximize relevance and engagement among NMTs working in the radiopharmacy cleanroom. The improvement observed in post-training knowledge scores, particularly in domains related to hygiene and radioactivity, suggests a positive mid-term educational impact. The absence of improvement in the radiopharmacy cleanroom and GRP scores may indicate the need for a particular emphasis on these topics. Nevertheless, scenario-based training should not be viewed as a standalone intervention. Its effectiveness is likely to be enhanced when integrated into a broader and coherent training strategy encompassing other key stages and interfaces of NM practice [48]. In particular, extending scenario-based approaches to radiopharmaceutical administration, patient identification, and interprofessional communication could further strengthen safety across the entire patient care pathway [49]. Complementary simulation-based training is also useful for acquiring and maintaining the preparation skills required for the production of radiopharmaceuticals [50,51]. Such integration aligns with contemporary patient safety models that emphasize system-based interventions rather than isolated corrective measures.

Beyond the local scope of this study, important perspectives emerge in terms of institutional and inter-site integration. Systematic reviews of PRIs combined with shared scenario-based training programs across multiple NM centers could promote collective learning, facilitate benchmarking, and contribute to the progressive harmonization of practices [52]. Learning from the working habits of other centers may also help identify latent risks that remain invisible at the single-center level. Embedding such initiatives within institutional quality management systems and continuing professional development frameworks could further enhance sustainability and reinforce safety culture [53].

Several limitations of this study should be acknowledged. Its monocentric design and the small number of participants in the training program limit the generalizability of the results and the extrapolation of knowledge questionnaire outcomes. In addition, the number of reported PRIs reflects only a portion of incidents that actually occurred. Underreporting, which is common in voluntary reporting systems, depends on safety culture, risk perception, training, and organizational constraints, leading to representativeness bias. Data quality was sometimes limited by incomplete or imprecise reports, requiring clarification from witnesses. Although the reclassification process and criticality assessment were structured and multidisciplinary, they inevitably involved a degree of subjectivity. Finally, the training scenarios were directly derived from local PRIs and reflect specific organizational practices, which may limit immediate transferability to other centers despite adherence to GMPs. Regarding the impact of the training, it would be of interest in the future to monitor the number of reported PRIs related to the topics covered, in order to gain insight into training retention over the medium and long term.

Despite these limitations, this study demonstrates the value of combining systematic PRI analysis with scenario-based training to enhance safety in NM and radiopharmacy practice. When integrated into institutional quality and safety strategies, such approaches may contribute to sustained improvements in professional practice and patient safety.

## 5. Conclusions

This work allowed us, first, to establish a detailed overview of adverse events occurring within the nuclear medicine department, highlighting the predominance of dysfunctions related to the medication-use process and identifying the most critical areas of the department. Second, it led to the development and implementation of an interactive, scenario-based training program specifically targeting these identified vulnerabilities.

The evaluation, based on questionnaire outcomes and qualitative participant feedback, demonstrated a significant improvement in operators’ knowledge. These findings underscore the value of a contextualized educational approach in strengthening the safety of professional practices. They also open perspectives for further expansion of the training program, notably through its integration into the continuing education curriculum for nuclear medicine technologists of our unit, its possible adaptation to other nuclear medicine settings, and the implementation of monitoring indicators to assess its real impact on daily practice and, ultimately, on the reduction in PRIs.

## Figures and Tables

**Table 1:** Criticality matrix used to classify PRIs as low (green)-, moderate (yellow)-, high (orange)-, or critical (red)-risk. Criticality levels were defined as follows: 1–4: low (routine monitoring); 5–9: moderate (planning of preventive actions); 10–16: high (immediate actions to mitigate risks); 20–25: critical (urgent measures to prevent serious consequences).

	Severity 1	Severity 2	Severity 3	Severity 4	Severity 5
Probability 1	1	2	3	4	5
Probability 2	2	4	6	8	10
Probability 3	3	6	9	12	15
Probability 4	4	8	12	16	20
Probability 5	5	10	15	20	25

**Table 2:** Distribution of the number of PRIs by the activity sector.

Activity Sector	Number of PRIs	Proportion of PRIs (%)
SPECT	116	52.0
PET	92	41.3
Therapy	14	6.3
Others	1	0.4

**Table 3:** Duration and content of scenario-based videos.

Topic of the Video	Duration	Errors to Identify
Failure to comply with hygiene and gowning protocols when entering the laboratory	53 s	Entry into the radiopharmacy cleanroom while the air-handling unit was not in operation. Failure to check the pressure indicators of the gowning airlock and the cleanroom. Non-compliance with handwashing procedures prior to gowning. Failure to follow the correct gowning sequence. Wearing jewelry. Absence of a passive finger dosimeter.
Handling of the ^99^Mo/^99m^Tc generator without adherence to good practice guidelines	1 m 03 s	Failure to reposition the generator cap after elution. Selection of an incorrect calibration factor on the dose calibrator (syringe factor selected while measuring a vial). Erroneous modification of the eluate vial volume in the information system (6 mL instead of 5 mL). Eluate vial shields not labeled immediately after measurement, increasing the risk of identification errors.
Preparation of radiopharmaceuticals incorporating errors commonly observed in the laboratory	41 s	Failure to verify the vial expiration date prior to use. Failure to disinfect the vial septum after removal of the flip-off cap.
Label printer failure leading to breaches of asepsis and hygiene within the shielded cell	39 s	Opening of the shielded cell while radioactive handling was in progress. Resumption of activity without bio-cleaning of the shielded cell after opening.
Preparation of patient-specific syringes without compliance with good handling practices	45 s	Sampling by vial inversion, with a risk of contamination of the shielded container and gloves. Reuse of the same saline bag to dilute several different radiopharmaceuticals. Sterile field positioned incorrectly, with the absorbent side not facing upward. Batch labeling of syringes rather than immediate labeling after measurement, increasing the risk of identification errors.
Dispensing of the quality control syringe and patient-specific syringes in the transfer hatch without adherence to dispensing procedures	27 s	Failure to clean the pass-through and absence of a sterile field. Presence of multiple syringes in the pass-through. Syringe arrangement compromising operator radiation protection. Dispensing of a quality-control syringe without a syringe shield and placement in the patient syringe pass-through.
Inadequate reception and dispensing of iodine-131 capsules	1 m 16 s	Incorrect electronic receipt of a capsule, with a discrepancy between the activity expected in the information system and the activity actually received. Electronic dispensing of the capsule based on an incorrect receipt, resulting in inaccurate electronic traceability.

## Data Availability

The original contributions presented in this study are included in the article/Supplementary Materials. Further inquiries can be directed to the corresponding author.

## Supplementary Materials

The following supporting information can be downloaded at: https://zenodo.org/records/18266979 (accessed on 5 February 2026), Video S1: Failure to comply with hygiene and gowning protocols when entering the laboratory. Video S2: Handling of the ^99^Mo/^99m^Tc generator without adherence to good practice guidelines. Video S3: Preparation of radiopharmaceuticals incorporating errors commonly observed in the laboratory. Video S4: Labeling errors leading to compromised asepsis and hygiene within the negative-pressure area. Video S5: Preparation of patient-specific syringes without compliance with good handling practices. Video S6: Dispensing of the quality control syringe and patient-specific syringes in the transfer hatch without adherence to dispensing procedures. Video S7: Inadequate reception and dispensing of iodine-131 capsules. File S1: Training questionnaire. File S2: Satisfaction survey.
